# Hydrotalcite framework stabilized ruthenium nanoparticles (Ru/HTaL): efficient heterogeneous catalyst for the methanolysis of ammonia-borane

**DOI:** 10.3906/kim-1910-44

**Published:** 2020-04-01

**Authors:** İsmail Burak BAĞUÇ, Mehmet YURDERİ, Gülşah SAYDAN KANBEROĞLU, Ahmet BULUT

**Affiliations:** 1 Department of Chemistry, Faculty of Science, Van Yüzüncü Yıl University, Van Turkey

**Keywords:** Ammonia-borane, methanolysis, hydrotalcite, ruthenium, nanoparticles

## Abstract

Ruthenium nanoparticles stabilized by a hydrotalcite framework (Ru/HTaL) were prepared by following a 2-step procedure comprising a wet-impregnation of ruthenium(III) chloride precatalyst on the surface of HTaL followed by an ammonia-borane (NH_3_BH_3_) reduction of precatalyst on the HTaL surface all at room temperature. The characterization of Ru/HTaL was done by using various spectroscopic and visualization methods including ICP-OES, P-XRD, FTIR, ^11^B NMR, XPS, BFTEM, and HRTEM. The sum of the results gained from these analyses has revealed the formation of well-dispersed and highly crystalline ruthenium nanoparticles with a mean diameter of 1.27 ±0.8 nm on HTaL surface. The catalytic performance of Ru/HTaL in terms of activity, selectivity, and stability was investigated in the methanolysis of ammonia-borane (NH_3_BH_3_ , AB), which has been considered as one of the most promising chemical hydrogen storage materials. It was found that Ru/HTaL can catalyse methanolysis of AB effectively with an initial turnover frequency (TOF) value of 392.77 min^-1^ at conversion (>95%) even at room temperature. Moreover, the catalytic stability tests of Ru/HTaL in AB methanolysis showed that Ru/HTaL acts as a highly stable and reusable heterogeneous catalyst in this reaction by preserving more than 95% of its initial activity even at the 5th recycle.

## 1. Introduction

One of the most critical obstacles in ‘hydrogen economy’ is the safe and efficient storage and release of hydrogen under mild conditions [1,2]. At this concern, the recent studies performed in this field have revealed that ammonia-borane (NH_3_BH_3_ , AB) needs serious consideration as one of the most promising solid materials in chemical hydrogen storage owing to its high hydrogen content (19.6 wt%), stability, and nontoxicity [3–5]. Hydrogen (H_2_) gas can be released from AB throughout its catalytic hydrolysis [6,7], alcoholysis [8–10], dehydrocoupling [11,12], and thermolysis [13-15]. Among these protocols the catalytic methanolysis of AB has remarkable advantages in term of both reaction kinetics and recycling of side product ammonium tetramethoxyborate (NH_4_ .B(OCH_3_)_4_) yielded along with H_2_ gas [16]. To date, various catalytic systems such as polymer stabilized-nickel(0) [17], Ru/MMT [18], PVP-stabilized Ru(0) [19], Co_48_Pd_52_ /C [20], Cu-Cu_2_ OCuO/C [21], mesoporous CuO [22], Rh(0)/nano-SiO_2_ [23], and copper nanoparticles [24] have been designed and tested in this important catalytic transformation, but the development of highly active and stable catalytic material is still one of the most important goals for this important catalytic transformation.

In this study we fabricated ruthenium(0) nanoparticles supported on a hydrotalcite framework (Ru/HTaL) by a 2-step procedure including wet-impregnation of ruthenium precatalyst and their borohydride reduction. Hydrotalcite (HTaL) is a layered anionic clay which is indicated as [M^2+^_1-x_ M^3+^_x_ (OH)_2_]^x+^A^n-1^_x/n_ •mH_2_ O. Here, M^2+^ and M^3+^ are di- and trivalent metal ions, and An^-^ is the anion between the layers [25,26]. Because of its (i) high adsorption capacity, (ii) high surface area, (iii) anion-exchange ability of the interlayer space, and (iv) high chemical stability, HTaL has drawn much attention as a support material in catalysis [27–30]. Hydrotalcite-supported Pt [31], Pd [32–34], Ru [35], and Au [36] nanoparticles have been used as catalysts in hydrogenation, oxidation, and coupling reactions. The characterization of Ru/HTaL has been done by using various spectroscopic and visualization techniques including inductively coupled plasma optical emission spectroscopy (ICP-OES), powder X-ray diffraction (P-XRD), Fourier transform infrared spectroscopy (FTIR), ^11^B NMR spectroscopy, X-ray photoelectron spectroscopy (XPS), scanning electron microscopy (SEM), bright field transmission electron microscopy (BFTEM), and high resolution transmission electron microscopy (HRTEM). The sum of the results gathered from these analyses has showed the formation of well-dispersed and highly crystalline ruthenium(0) nanoparticles on the surface of HTaL. The catalytic employment of Ru/HTaL has been done on the catalytic methanolysis of AB (Eq 1) and the catalytic performance of Ru/HTaL has been investigated in terms of activity and stability provided in the methanolysis of AB. We found that Ru/HTaL can effectively catalyse the methanolysis of AB with an initial turnover frequency (TOF) value of 392.77 min^-1^ at complete conversion (>99%) and at room temperature. The stability of Ru/HTaL was also investigated by performing recyclability experiments, which pointed out that Ru/HTaL is a highly stable catalytic material in the methanolysis of AB by preserving almost its inherent activity even at the 5th recycle.

(1)NH3BH3+4CH3OH→NH4B(OCH3)4+3H2

## 2. Materials and methods

### 2.1. Chemicals

Ruthenium(III) chloride trihydrate (RuCl_3_ .3H_2_ O), ammonia-borane (NH_3_BH_3_ ~97%), magnesium nitrate hexahydrate (Mg(NO_3_) .6H_2_ O), aluminium nitrate nonohydrate (Al(NO_3_)_3_ •9H_2_ O), ethanol (C_2_H_5_ OH), sodium hydroxide (NaOH), sodium carbonate (Na_2_CO_3_), and methanol (CH_3_OH) were purchased from Sigma-Aldrich. Methanol was distilled over magnesium (Mg) and stored in a Schlenk tube under argon atmosphere. Distilled water used in all experiments was provided from the Milli-Q water purification system. All glassware and Teflon materials were washed with the acetone/water mixture and dried in an oven at 150 °C.

### 2.2. Characterization

Ruthenium amount deposited on the surface of Ru/HTaL was estimated by ICP-OES analyses performed on ULTIMA 2-HORIBA Jobin-Yvon by dissolving Ru/HTaL samples in a HCl/HNO_3_ mixture. P-XRD patterns were taken from a Rigaku Ultima-IV P-XRD device (Cu-Kα radiation wavelength 1.54051 Å, 30 kV, 15 mA). FTIR spectra was taken from KBr pellet on a Shimadzu IR Affinity1 spectrophotometer. ^11^B NMR spectra were recorded on a Bruker Avance DPX 400 with an operating frequency of 128.15 MHz for ^11^B.D_2_ O and BF_3_•(C_2_H_5_)_2_ O used as a lock and an external reference, respectively. BFTEM images were taken from the JEOL JEM-200CX instrument operating at 120 kV and the high-resolution electron microscope (HRTEM) images were taken from the JEOL JEM-2010F instrument operating at 200 kV. XPS analyses of Ru/HTaL were carried out using a Kratos AXIS XPS spectrometer equipped with monochromatic Al-Kα radiation (1486.6 eV, 15 kV, 350 W) with transition energy of 23.5 eV.

### 2.3. Synthesis of hydrotalcite (HTaL)

Synthesis of HTaL support material was carried out by following a procedure previously reported in the literature [37]. In a typical synthesis protocol, an aqueous solution containing 28.78 mmol (7.38 g) magnesium nitrate hexahydrate and 14.4 mmol (5.40 g) aluminium nitrate nonohydrate solutions was mixed with sodium hydroxide (1.0 M) and sodium carbonate (0.4 M) solutions at about pH 10.0 (±0.1). The resulting mixture was stirred for 6 h at 353 K and then centrifuged at 6000 rpm for 10 min to isolate the solid part of the aliquot. The isolated solid was washed with distilled water until a pH value of around 7.0 was reached in aliquot taken from the liquid part of the filtrate. The resulting solid product (HTaL) was dried at 383 K for 12 h.

### 2.4. Preparation of Ru/HTaL catalyst

As aforementioned the Ru/HTaL catalyst was prepared by following a 2-step procedure comprising wetimpregnation of RuCl_3_ .3H_2_ O precatalyst followed with its borohydride reduction. For this purpose, methanol solution (5.0 mL) of ruthenium (29.9 μmol Ru; 7.84 mg RuCl_3_ .3H_2_ O) was stirred with HTaL (150 mg) in a small beaker at 400 rpm for 3 h. Then, 1.0 mL of aqueous AB (14.61 mg NH_3_BH_3_ , 0.47 mmol) solution was added to this mixture to achieve the reduction of ruthenium precatalyst (Ru^3+^→ Ru^0^) . Next, the solid reaction products (Ru/HTaL) were isolated through centrifugation (6000 rpm, 5 min) washed with ethanol (3 ×20 mL), and dried in a vacuum-oven for 12 h at 150 °C and 0.1 bar vacuum.

### 2.5. Testing the catalytic activity of Ru/HTaL in the methanolysis of AB

The catalytic activity of Ru/HTaL in the methanolysis of AB was determined by measuring the hydrogen generation rate. The volume of the gas released during the reaction was monitored by using a gas burette water displacement as reported in the literature [38–40].

First, the desired amount of Ru/HTaL catalyst was weighed and taken into the jacketed Schlenk tube, which was thermostated through a water-circulation system to maintain constant temperature, containing 4.0 mL of methanol. Then, the resulting solution was mixed at 600 rpm for 15 min to achieve thermal equilibrium. Next, 1.0 mL of methanol solution of 0.51 mmol AB (15.91 mg) was added to the reaction flask rapidly through the septum in the upper part of the flask by using a 1.0-mL gas-tight syringe and the catalytic reaction started. The same methodology was followed in the investigation of (i) effect of catalyst concentration, (ii) effect of substrate concentration, and (iii) effect of temperature on the rate of the catalytic reaction.

### 2.6. The effect of temperature on the rate of Ru/HTaL catalysed methanolysis of AB

To investigate the effect of temperature on the rate of Ru/HTaL (50 mg, 1.12 wt% Ru) catalysed methanolysis of AB (100 mM in 5.0 mL of methanol), the catalytic reaction was performed at different temperatures (25, 30, 35, 40, and 45°C). The initial rates at each temperature were determined from the linear portion of plots (volume of H_2_ versus time) and used for the construction of Arrhenius and Eyring plots [41,42] from where activation parameters were determined.

### 2.7. The effect of substrate concentration on the rate of Ru/HTaL catalysed methanolysis of AB

To examine the effect of substrate concentration ([AB]) on the rate of Ru/HTaL (50 mg, 1.12 wt% Ru) catalysed methanolysis of AB (in 5.0 mL of methanol), the catalytic reaction was performed at 35 °C starting with various AB concentrations (50, 75, 100, 125, and 150 mM). The initial rates at each AB concentration were determined from the linear portion of plots (volume of H_2_ versus time) and used for the construction of the lnkobs versus ln[AB] graph.

### 2.8. The effect of catalyst ruthenium concentration on the rate of Ru/HTaL catalysed methanolysis of AB

To inspect the effect of catalyst concentration ([Ru]) on the rate of Ru/HTaL catalysed methanolysis of AB (100 mM in 5.0 mL of methanol), the catalytic reaction was performed at 35 °C starting with various Ru concentrations (0.55, 1.11, 2.22, and 4.44 mM). The initial rates at each Ru concentration were determined from the linear portion of plots (volume of H_2_ versus time) and used for the construction of the lnkobs versus ln[Ru] graph.

### 2.9. Testing the recyclability performance of Ru/HTaL catalyst in the methanolysis of ammoniaborane

The recyclability performance of Ru/HTAL in the methanolysis of AB was determined by performing a series of experiments that were started with 5.0 mL of methanol solution (4.0 mL of methanol + 1.0 mL of methanol solution with 0.51 of mmol AB) at 35 °C. When the first run finished at complete conversion, another equimolar amount of fresh substrate (AB) was added to the reaction mixture immediately and the same procedure was followed up to the 5th catalytic recycling.

## 3. Results

### 3.1. Characterization of Ru/HTaL

**Figure 1:**
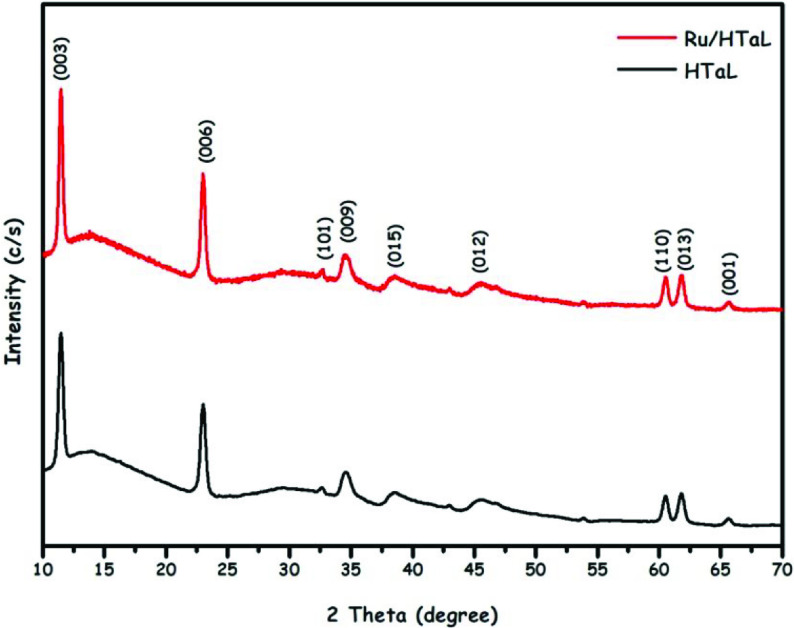
The P-XRD pattern of the prepared hydrotalcite nanoparticles taken between (HTaL) 2θ = 10–70°.

Ru/HTaL catalyst was prepared by following a 2-step procedure comprising wet-impregnation of ruthenium(III) chloride precatalyst on the surface of HTaL followed by AB reduction of precatalyst on HTaL surface all at room temperature. The solid Ru/HTaL product was then isolated to carry out characterization studies. Firstly, to determine the amount of ruthenium loading in the Ru/HTaL sample, ICP-OES analyses were carried out, which showed that the Ru/HTaL sample prepared with our protocol contains 1.12 wt% ruthenium. The crystallinities of the host (HTaL) and Ru/HTaL materials were checked by P-XRD studies and P-XRD patterns of these 2 materials are depicted in Figure 1. The comparison of these 2 P-XRD patterns clearly shows that the crystallinity of the host material (HTaL) was retained at the end of the synthesis protocol as the definite Bragg peaks of HTaL are seen in the P-XRD pattern of the Ru/HTaL material. It should also be noted that Bragg peaks of metallic ruthenium were not observed in the P-XRD pattern of Ru/HTaL due to the low level of ruthenium loading [43]. We also performed FTIR analyses on HTaL, Ru(III)/HTaL, and Ru(0)/HTaL (Figure S1, Supplementary Information), which indicate that the surface functionality of HTaL remains intact with the synthesis protocol.

**Figure 2:**
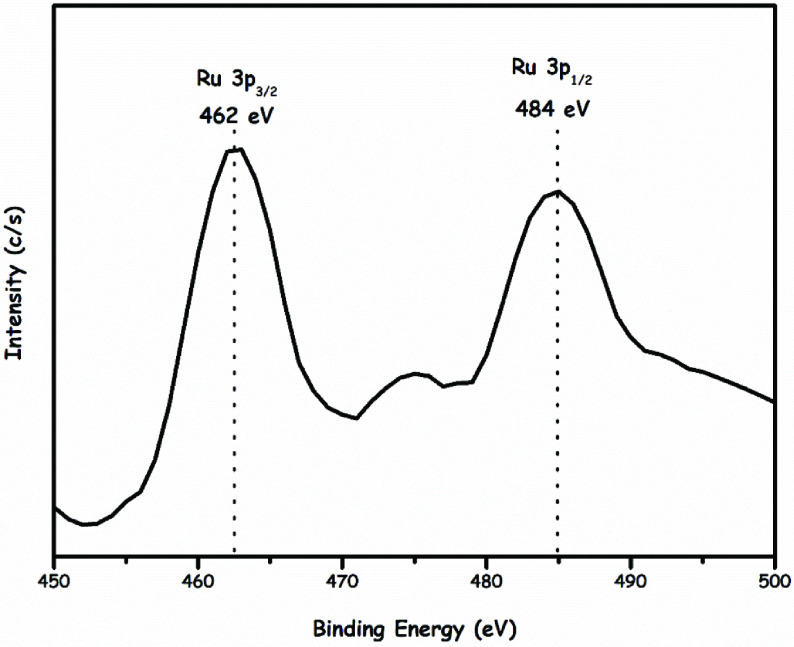
Ru 3p region (450-500 eV) high-resolution XPS spectrum of hydrotalcite framework stabilized ruthenium nanoparticles (Ru/HTaL).

**Figure 3:**
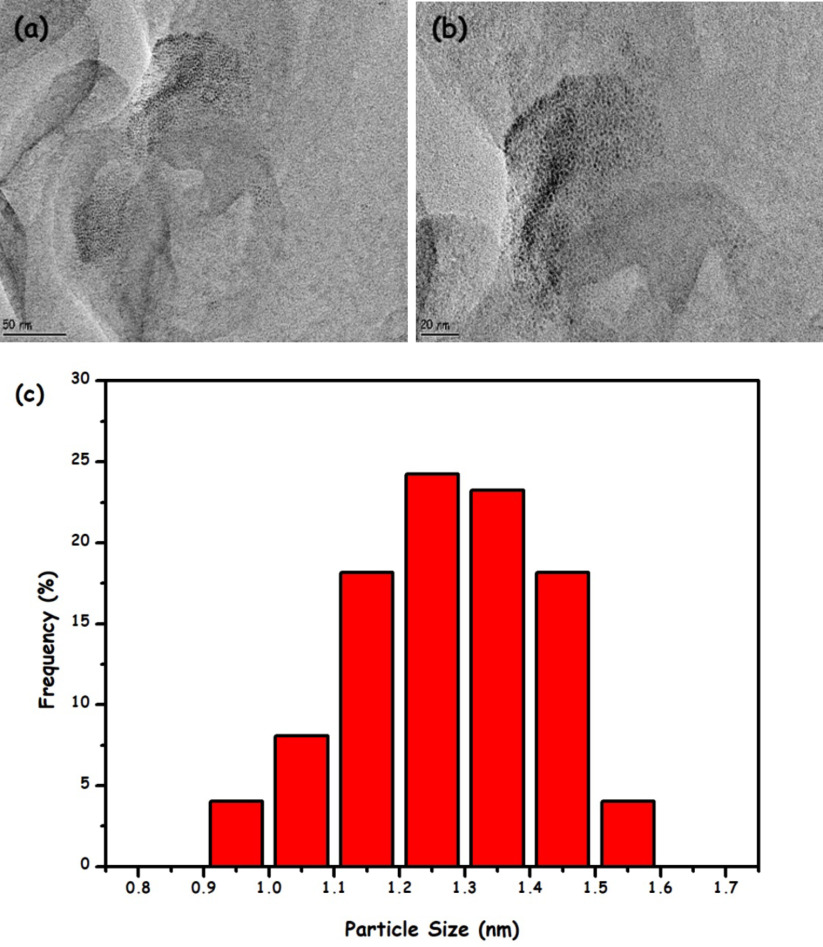
(a–b) TEM images Ru/HTaL in different magnifications and (c) size histogram of hydrotalcite framework stabilized ruthenium nanoparticles (Ru/HTaL).

**Figure 4:**
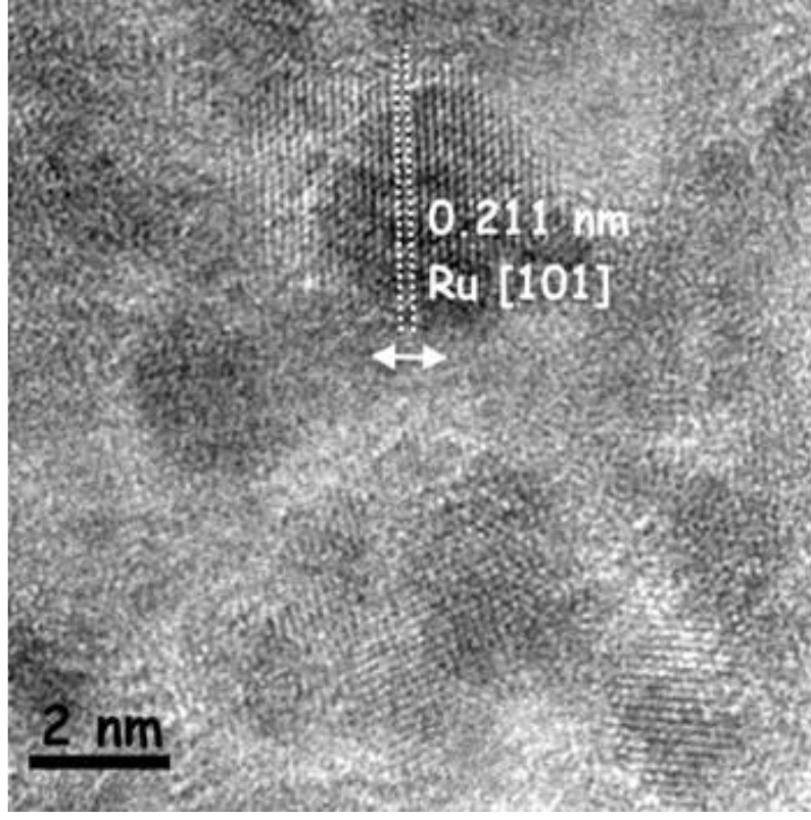
A high-resolution TEM image of Ru/HTaL.

Then, to investigate the oxidation state of ruthenium in the Ru/HTaL catalyst, XPS analysis was performed on the Ru/HTaL sample. The high resolution XPS spectrum of Ru/HTaL taken at Ru 3p core level is given in Figure 2, which shows 2 distinct peaks at 462 and 484 eV that can be assigned to Ru 3p_3/2_ and 3p_1/2_ bands of metallic ruthenium [44]. In order to examine the size, morphology, and crystallinity of the resulting ruthenium(0) nanoparticles in the Ru/HTaL catalyst, TEM and HRTEM analyses were done on Ru/HTaL. TEM images of the Ru/HTaL given in Figures 3a and 3b clearly show the formation of highly dispersed ruthenium(0) nanoparticles with an average particle size of 1.27 ±0.8 nm (Figure 3c), which was found by counting 85 particles with the ImageJ program [45] and there is no agglomeration and clumping of ruthenium(0) nanoparticles on the surface of HTaL. Additionally, the crystallinity of the resulting HTaLsupported ruthenium nanoparticles was also inspected by electron microscopy. The HRTEM image of Ru/HTaL given in Figure 4 indicates a highly crystalline feature. The crystalline fringe distance of ruthenium nanoparticle was measured to be 0.21 nm, which corresponds to the Ru (101) surface [46,47,48].

### 3.2. Investigation of the catalytic performances of hydrotalcite framework stabilized ruthenium nanoparticles (Ru/HTaL) in the methanolysis of ammonia-borane

**Figure 5:**
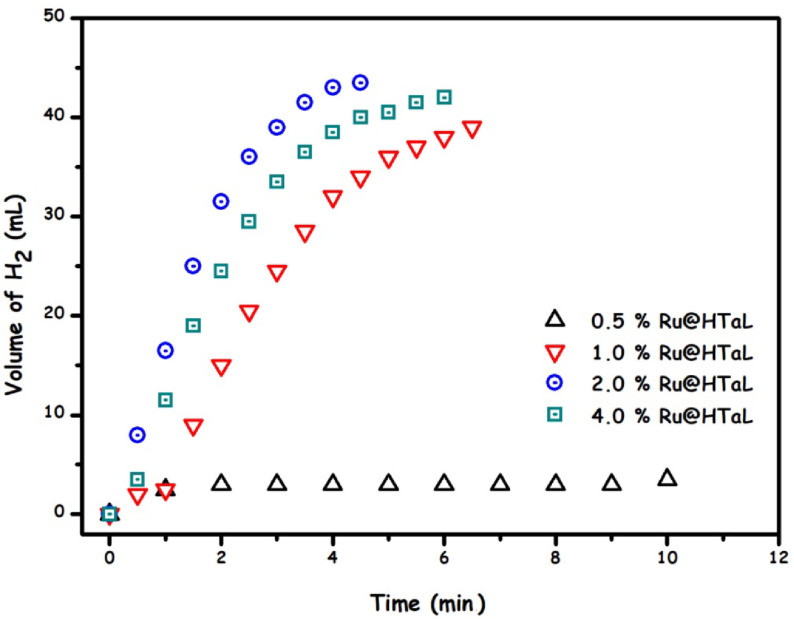
The concentration of Ru/HTaL with different wt% Ru loading.

Before examining the catalytic activity of the resulting ruthenium(0) nanoparticles in the Ru/HTaL catalyst, the catalytic reactivity of ruthenium-free host material HTaL was checked in the methanolysis of AB, and it was found that HTaL material was catalytically inactive in this reaction. Then, the catalytic activity of Ru/HTaL materials with different Ru% loadings were tested in the methanolysis of AB at the same ruthenium concentrations ([Ru]) and 35 °C. The results of these catalytic experiments are presented in Figure 5 as the plot of the volume of hydrogen gas (H_2_) generated versus time. This graph clearly indicates that the highest activity can be achieved in the presence of Ru/HTaL catalyst that contains 1.12 wt% Ru. For that reason, in all experiments reported hereafter, Ru/HTaL with 1.12 wt% Ru loaded is used. In addition to the volumetric hydrogen gas measurement, we also performed ^11^B NMR studies. The ^11^B NMR spectrum of reaction (Figure S2, Supplementary Information) solution taken at the end of the catalytic reactions showed that NH_3_BH_3_ (δ = –24 ppm, q) was completely converted to NH_4_ B(OMe)4 (δ = 8.9 ppm, s). It should also be noted that most of the ruthenium nanoparticles in Ru/HTaL with 0.5% wt Ru loading exist on the defect sites of the host framework, which cannot be simply reached by substrate molecules. This is the main reason why we observed the lowest activity by this catalytic system even though it has the same ruthenium concentration as those of Ru/HTaL samples with different ruthenium loadings [49].

**Figure 6:**
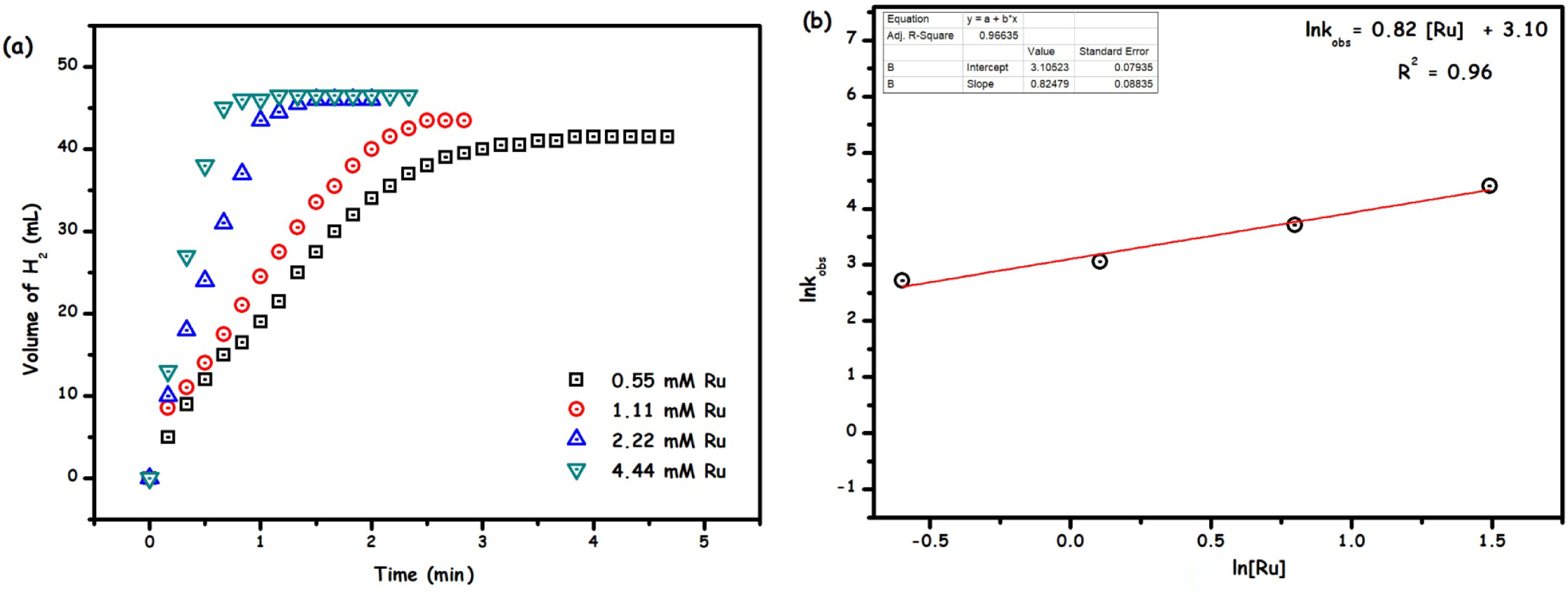
(a) The volume of H2 versus time plot for the methanolysis of ammonia-borane catalysed Ru/HTaL at the different ruthenium concentration and (b) plot of H2 generation rate versus the ruthenium concentration in logarithmic scale (rate = mL of H2 /min; [AB] = 100 mM, T = 35 °C).

The effect of the catalyst concentration ([Ru]) on Ru/HTaL catalysed methanolysis of the AB reaction was investigated by performing a series of experiments in which ruthenium concentration was varied ([Ru] = 0.55, 1.11, 2.22, and 4.44 mM) at constant AB concentration and 35 °C. Figure 6a shows the plot of the volume of hydrogen (H_2_) generated versus time graph gathered from these experiments. Expectedly, the increase in ruthenium concentration enhances the rate of hydrogen generation. The initial rates determined from the linear part of each slope were used to construct the lnkobs versus ln[Ru] graph given in Figure 6b, whose slope was found to be almost 1, indicating that the reaction proceeds in the first order with respect to ruthenium concentration.

**Figure 7:**
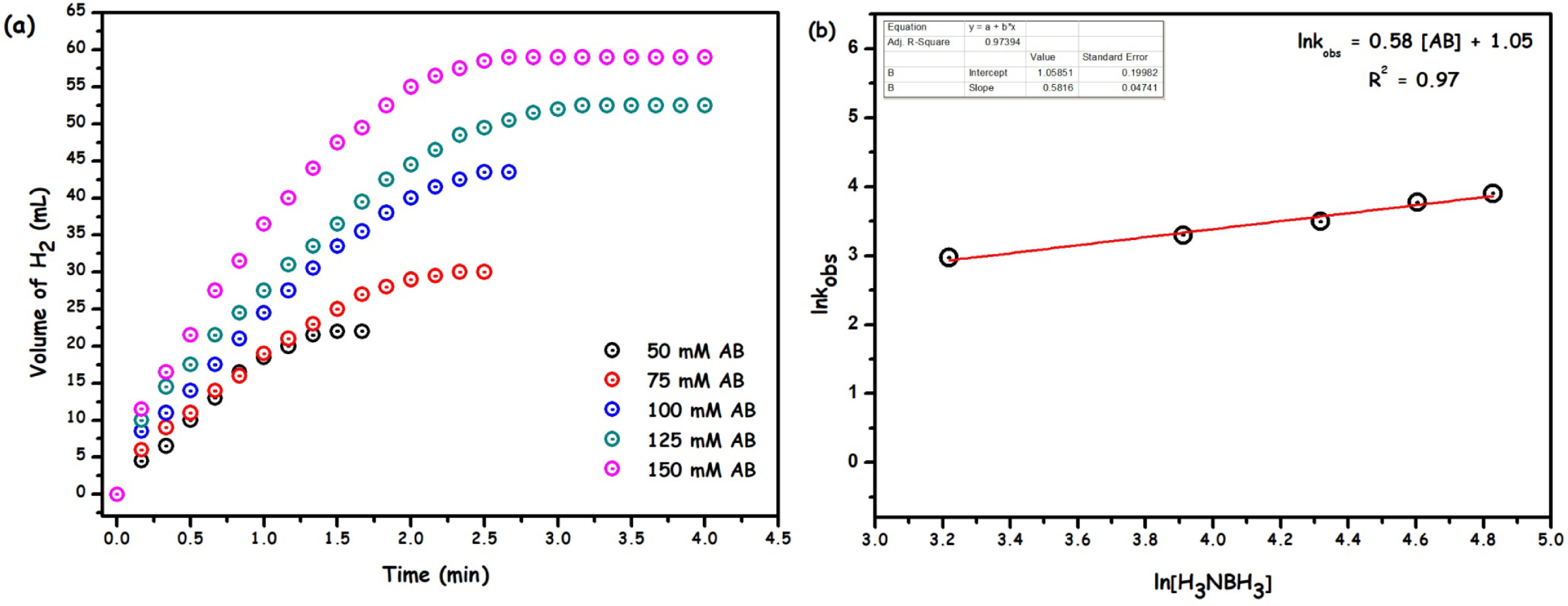
(a) The volume of H2 versus time plot for the methanolysis of ammonia-borane catalysed Ru/HTaL at the different ammonia-borane concentration and (b) plot of H2 generation rate versus the ammonia-borane concentration in logarithmic scale (rate = mL of H2 /min; [AB] = 100 mM, T = 35 °C).

Next, the effect of substrate concentration [AB] on the rate of the catalytic reaction was investigated by performing experiments in a similar manner as aforementioned, in which the starting concentration of AB was varied (50, 75, 100, 125, and 150 mM) at constant catalyst concentration ([Ru] = 1.11 mM) and temperature (35 °C). Figure 7a shows the plot of the amount of the released hydrogen gas (H_2_) versus time. As one can see, the reaction rate increases as the AB concentration increases and the initial rates were also determined from the linear portions of each slope and transformed into the lnkobs versus ln[AB] graph depicted in Figure 7b, whose slope was found to be 0.58, indicative of the reaction proceeding in half-order with respect to AB concentration.

**Figure 8:**
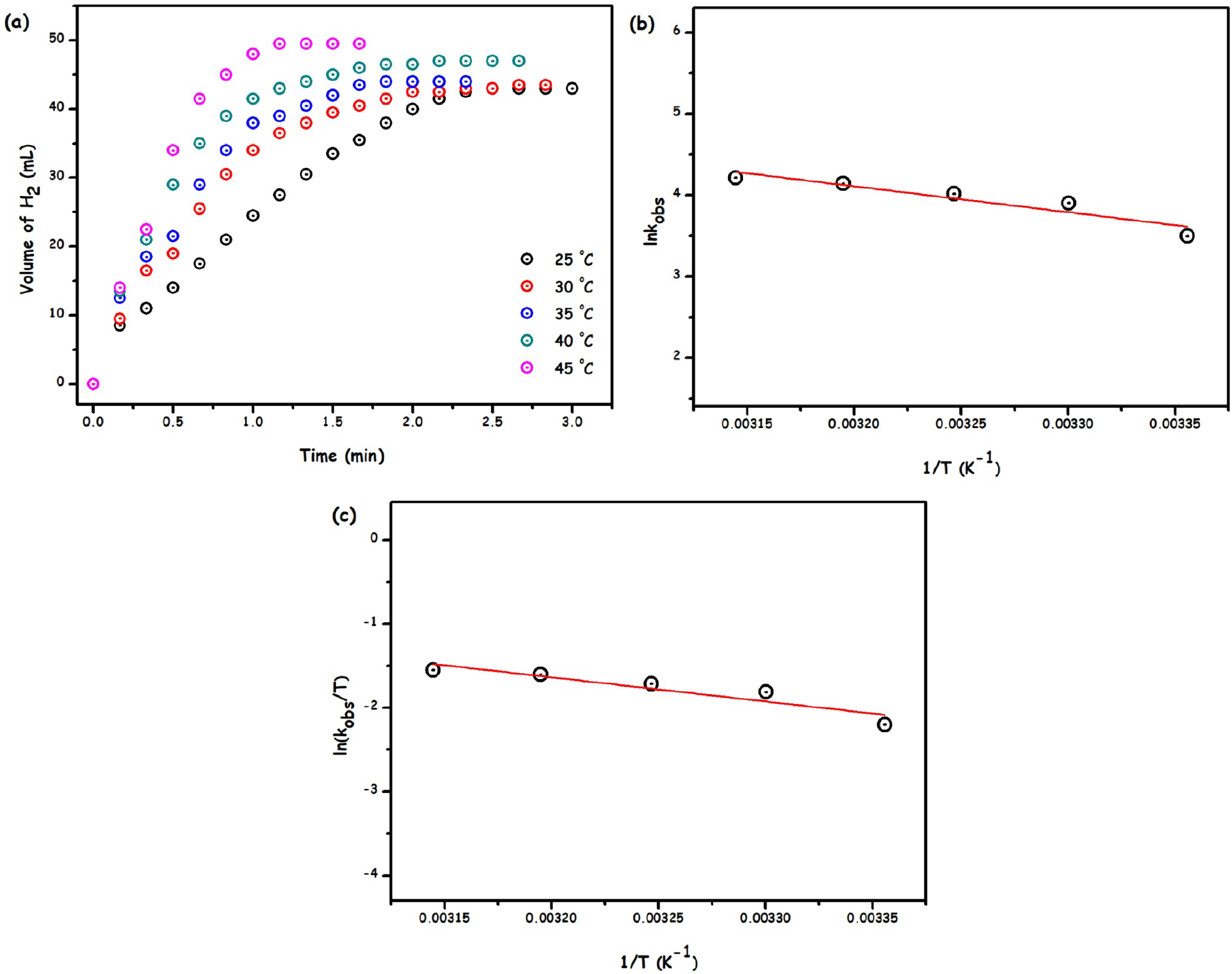
(a) The volume of H_2_ versus time plot for the methanolysis of ammonia-borane catalysed Ru/HTaL at the different temperatures, (b) Arrhenius plot of ln k versus 1/T ([AB] = 100 mM, [Ru] = 1.11 mM), and (c) Eyring–Polanyi equation plot of ln (k/T) versus 1/T ([AB] = 100 mM, [Ru] = 1.11 mM)

In addition to the effect of catalyst and substrate concentrations, the effect of temperature on the rate of Ru/HTaL catalysed methanolysis of AB was also investigated by performing the catalytic reaction at different temperatures (25, 30, 35, 40, 45 °C) and constant [Ru] and [AB] concentrations. As expected, the rate of the catalytic reaction was enhanced by the increase of temperature and the initial rates were determined from the linear part of each slope at different temperatures (Figure 8a) to construct Arrhenius (Figure 8b) and Eyring (Figure 8c) plots in order to determine activation parameters (Ea , ΔH#, ΔS#) of the catalytic reaction. The Arrhenius plot given in Figure 8b gives an activation energy value (E_a_) of 25.51 kJ/mol for the Ru/HTaL catalysed methanolysis of AB, and this activation energy value is lower than those of the catalytic system previously reported for the methanolysis of AB such as zeolite confined Rh (Ea = 40 ±2 kJ mol^-1^) [8], Ru/MMT (Ea = 23.8 kJ mol^-1^) [18], Co_48_Pd_52_ /C (Ea = 25.5 kJ mol^-1^) [20], Cu-Cu_2_ O-CuO/C (Ea = 67.9 kJ mol^-1^) [21], Rh(0)/nanoHAp (E_a_ = 56 ±2 kJ mol^-1^) [50], Ru/graphene (Ea = 54 ±2 kJ mol^-1^) [51], PVP-stabilized Pd (E_a_ = 35 ±2 kJ mol^-1^) [52], and AgPd alloy (E_a_ = 37.5 kJ mol^-1^) [53]. The activation enthalpy (ΔH#) and entropy (ΔS#) values of Ru/HTaL catalysed AB methanolysis were determined from the Eyring plot and found to be 23.95 kJ/mol and –134.5 J/mol K, respectively. The negative ΔS# value is indicative of the associative mechanism that occurred in the transition state of Ru/HTaL catalysed methanolysis of AB.

**Figure 9:**
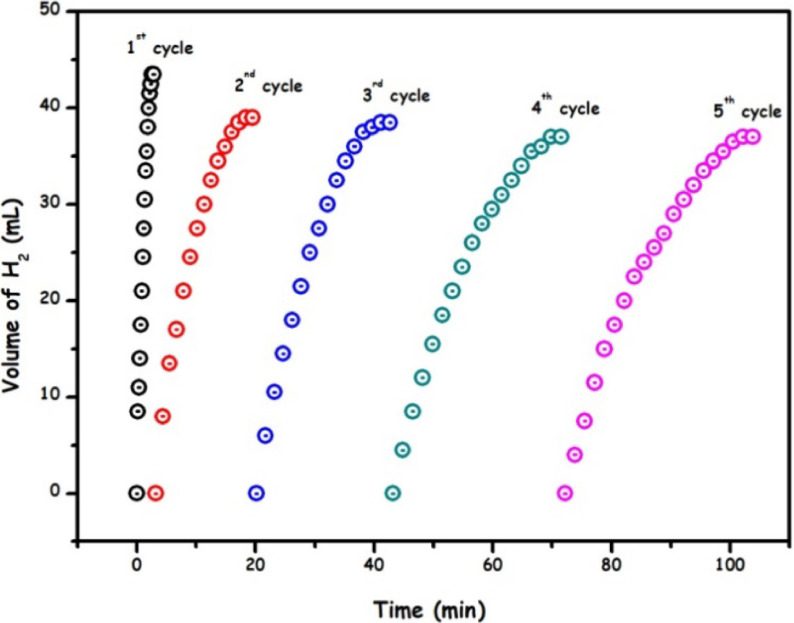
Recyclability performance graph up to 5th cycle for Ru/HTaL catalysed methanolysis of ammonia-borane.

Finally, the reusability performance of the Ru/HTaL catalyst was examined in the methanolysis of AB by carrying out Ru/HTaL catalysed AB methanolysis up to the 5th recycle, in which equimolar amount of fresh AB of each cycle was added at a rate of >90% conversion achieved in the previous run. The results of these experiments are given in Figure 9, which show that Ru/HTaL catalyst can retain more than 95% of its inherent activity even at the 5th recycle. These results are indicating that Ru/HTaL is a highly stable and reusable catalytic material in the methanolysis of AB.

## 4. Discussion

In this study, hydrotalcite framework stabilized ruthenium nanoparticles were prepared, characterized, and used as heterogeneous catalysts in the methanolysis of AB. The important findings of this study can be summarized as follows:

• Ruthenium(0) nanoparticles decorated on the hydrotalcite (HTaL) can be simply prepared by a 2-step procedure including wet-impregnation followed with a chemical reduction technique,

• The characterization of the resulting Ru/HTaL material was done by using various spectroscopic and visualization techniques, which showed the formation of highly dispersed and crystalline ruthenium(0) nanoparticles with a mean diameter of 1.27 ±0.8 nm on the surface of HTaL,

• Ru/HTaL was found to be an active catalyst in the methanolysis of AB, which provides a TOF value of 392.77 min^-1^ at 25 °C. This activity value is highly notable by comparing the previous catalytic systems reported for this catalytic reaction (Table),

**Table:** Values of TOF for the methanolysis of AB by various catalyst (25 °C).

Catalyst	TOF (mol H_2_ mol catalyst^-1^ min^-1^)	Recycle	Reference
Zeolite confined Rh	30.0	N.D.	[8]
RhCI_3_	100	N.D.	[16]
RuCI_3_	150	N.D.	[16]
PdCI_2_	1.5	N.D.	[16]
Pd/C	1.9	N.D.	[16]
MMT stabilized Ru	90.9	95% at 20th cycle	[18]
Co_48_Pd_52_/C	27.7	98% at 8th cycle	[20]
Rh(0)/nanosilica	168	N.D.	[23]
Rh(0)/nanoHAP	147	21% at 6th cycle	[50]
Ru/graphene	99.4	73.2% at 15th cycle	[51]
PVP-stabilized Pd	22.3	N.D.	[52]
Rh(0)/nanoalumina	218	N.D.	[54]
Rh/CC3-R-homo	215.3	N.D.	[55]
Cu/Co(OH)_2_	61.63	N.D.	[56]
Rh-PRO/C	1035	52% at 4th cycle	[57]
Ru/HTaL	392.77	95% at 5th cycle	This study

*N.D.: Not demonstrated

• The activation parameter (ΔH#, ΔS#) values of Ru/HTaL catalysed AB methanolysis were also determined and these values are suggestive of the associative mechanism for Ru/HTaL catalysed methanolysis of AB,

• The catalytic stability of Ru/HTaL in the methanolysis of AB was examined by carrying out recyclability experiments, which displayed that Ru/HTaL is a highly stable and recyclable catalyst for this important reaction by preserving >95% of its activity even at the 5th recycle.

Supplementary Materials

## References

[1] (2001). Zu\textasciidieresis ttel A. Hydrogen-storage materials for mobile applications. Nature.

[2] (2009). Schu\textasciidieresis th F. Chemical and physical solutions for hydrogen storage. Angewandhe Chemical International Edition.

[3] (2007). Will we soon be fueling our automobiles with ammonia-borane?. Angewandte Chemical International Edition.

[4] (2007). Ammonia-borane: the hydrogen source par excellence?. Dalton Transactions.

[5] (2008). Hydrogen-rich boron-containing materials for hydrogen storage. Dalton Transactions.

[6] (2019). Noble metal nanoparticles supported on activated carbon: Highly recyclable catalysts in hydrogen generation from the hydrolysis of ammonia borane. Journal of Colloid and Interface Science.

[7] (2019). Effect of LDH composition on the catalytic activity of Ru/LDH for the hydrolytic dehydrogenation of ammonia borane. International Journal of Hydrogen Energy.

[8] (2010). Zeolite confined rhodium(0) nanoclusters as highly active, reusable, and long-lived catalyst in the methanolysis of ammonia-borane. Applied Catalysis B: Environmental.

[9] (2017). Evenly dispersed microspherical amorphous alloy CoxB1-x: Robust and magnetically recyclable catalyst for alcoholyzing ammonia borane to release H$_{2}$. Materials Chemistry and Physics.

[10] (2016). Effect of morphology on catalytic performance of colloid Ru for hydrogen generation from H$_{3}$NBH$_{3}$ alcoholysis: a comparative study. Integrated Ferroelectrics.

[11] (2019). A highly Active bidentate magnesium catalyst for amine-borane dehydrocoupling: kinetic and mechanistic studies. Chemistry-A European Journal.

[12] (2017). -11 MASNMR study of the thermolytic dehydrocoupling of two ammonia boranes upon the release of one equivalent of H$_{2}$ at isothermal conditions. Chemistry Select.

[13] (2019). Discrepancy in the thermal decomposition/dehydrogenation of ammonia borane screened by thermogravimetric analysis. International Journal of Hydrogen Energy.

[14] (2018). Kinetic model analysis and mechanistic correlation of ammonia borane thermolysis under dynamic heating conditions. International Journal of Hydrogen Energy.

[15] (2017). Ammonia borane-boron composites for hydrogen release: thermolysis kinetics. Energy Sources.

[16] (2007). Preparation of ammonia borane in high yield and purity, methanolysis, and regeneration. Inorganic Chemistry.

[17] (2015). PVP-stabilized nickel(0) nanoparticles as catalyst in hydrogen generation from the methanolysis of hydrazine borane or ammonia borane. Applied Catalysis B: Environmental.

[18] (2010). Ruthenium nanoparticles immobilized in montmorillonite used as catalyst for methanolysis of ammonia borane. International Journal of Hydrogen Energy.

[19] (2011). Hydrogen generation from the methanolysis of ammonia borane catalyzed by in-situ generated polymer stabilized ruthenium(0) nanoclusters. Catalysis Today.

[20] (2012). Methanolysis of ammonia borane by CoPd nanoparticles. American Chemical Society Catalysis.

[21] (2015). Supported copper-copper oxide nanoparticles as active, stable and low-cost catalyst in the methanolysis of ammonia-borane for chemical hydrogen storage. Applied Catalysis B: Environmental.

[22] (2015). Methanolysis of ammonia borane by shape-controlled mesoporous copper nanostructures for hydrogen generation. Dalton Transaction.

[23] (2016). Rhodium(0) nanoparticles supported on nanosilica: highly active and long lived catalyst in hydrogen generation from the methanolysis of ammonia borane. Applied Catalysis B: Environmental.

[24] (2008). Nanostructured Cu and Cu@Cu$_{2}$O core shell catalysts for hydrogen generation from ammonia-borane. Physical Chemistry Chemical Physics.

[25] (1980). Physico-chemical properties of synthetic hydrotalcites in relation to composition. Clays Clay Minerals.

[26] (2008). Mg/Al ordering in layered double hydroxides revealed by multinuclear NMR spectroscopy. Science.

[27] (1991). Hydrotalcite-type anionic clays: preparation, properties and applications. Catalysis Today.

[28] (1999). Layered double hydroxides exchanged with tungstate as biomimetic catalyst for mild oxidative bromination. Nature.

[29] (2001). Hydrotalcite-like anionic clays in catalytic organic reactions. Catalysis Reviews Science and Engineering.

[30] (2009). Exploring, tuning, and exploiting the basicity of hydrotacites for applications in heterogeneous catalysis. Chemistry European Journal.

[31] (2013). A mild solution chemistry method to synthesize hydrotalcite-supported platinum nanocrystals for selective hydrogenation of cinnamaldehyde in neat water.

[32] (2015). Pd nanoparticles on hydrotalcite as an efficient catalyst for partial hydrogenation of acetylene: effect of support acidic and basic properties. Journal of Catalysis.

[33] (2006). Palladium and copper supported on mixed oxides derived from hydrotalcite as reusable solid catalysts for the Sonogashira coupling. Journal of Catalysis.

[34] (2018). The effect of the hydrotalcite structure and nanoparticle size on the catalytic performance of supported palladium nanoparticle catalysts in Suzuki cross-coupling. Applied Catalysis A: General.

[35] (2017). Nanohydrotalcite supported ruthenium nanoparticles: highly efficient heterogeneous catalyst for the oxidative valorization of lignin model compounds. Chemistry Select.

[36] (2019). Bio-based chemicals: selective aerobic oxidation of tetrahydrofuran-2,5-dimethanol to tetrahydrofuran-2,5-dicarboxylic acid using hydrotalcite-supported gold catalysts. ACS Sustainable Chemistry & Engineering.

[37] (2015). Facile and surfactant-free synthesis of supported Pd nanoparticles on hydrotalcite for oxidation of benzyl alcohol. Royal Society of Chemistry Advances.

[38] (2015). MnOx-promoted PdAg alloy nanoparticles for the additive-free dehydrogenation of formic acid at room temperature. ACS Catalysis.

[39] (2014). Carbon supported trimetallic PdNiAg nanoparticles as highly active, selective and reusable catalyst in the formic acid decomposition. Applied Catalysis B: Environmental.

[40] (2015). Amine grafted silica supported CrAuPd alloy nanoparticles: superb heterogeneous catalysts for the room temperature dehydrogenation of formic acid. Chemical Communications.

[41] (2016). PdAu-MnOx nanoparticles supported on amine-functionalized SiO$_{2}$ for the room temperature dehydrogenation of formic acid in the absence of additives. Applied Catalysis B: Environmental.

[42] (2017). Nanoceria supported cobalt(0) nanoparticles: a magnetically separable and reusable catalyst in hydrogen generation from the hydrolysis of ammonia borane. New Journal of Chemistry.

[43] (2018). Nanocrystalline metal organic framework (MIL-101) stabilized copper nanoparticles: Highly efficient nanocatalyst for the hydrolytic dehydrogenation of methylamine borane. Inorganica Chimica Acta.

[44] (2016). Metal-organic framework (MIL-101) stabilized ruthenium nanoparticles: highly efficient catalytic material in the phenol hydrogenation. Microporous and Mesoporous Materials.

[45] (2006). Analysis of nanoparticle transmission electron microscopy data using a public-domain image-processing program, image. Turkish Journal of Chemistry.

[46] (2018). Atomic layer deposition of ruthenium nanoparticles on electrospun carbon nanofibers: a highly efficient nanocatalyst for the hydrolytic dehydrogenation of methylamine borane. ACS Applied Materials & Interfaces.

[47] (2014). Ruthenium(0) nanoparticles stabilized by metal-organic framework (ZIF-8): Highly efficient catalyst for the dehydrogenation of dimethylamine-borane and transfer hydrogenation of unsaturated hydrocarbons using dimethylamine-borane as hydrogen source. Applied Catalysis B: Environmental.

[48] (2016). Highly selective hydrogenation of arenes using nanostructured ruthenium catalysts modified with a carbon-nitrogen matrix. Nature Communications.

[49] (1989). Structure and Reactivity of Surfaces.

[50] (2015). Rhodium(0) nanoparticles supported on hydroxyapatite nanospheres and further stabilized by dihydrogen phosphate ion: a highly active catalyst in hydrogen generation from the methanolysis of ammonia borane. International Journal of Hydrogen Energy.

[51] (2015). An improved preparation of graphene supported ultrafine ruthenium (0) NPs: Very active and durable catalysts for H$_{2}$ generation from methanolysis of ammonia borane. International Journal of Hydrogen Energy.

[52] (2009). In situ-generated PVP-stabilized palladium(0) nanocluster catalyst in hydrogen generation from the methanolysis of ammonia-borane. Physical Chemistry Chemical Physics.

[53] (2016). Monodisperse AgPd alloy nanoparticles as a highly active catalyst towards the methanolysis of ammonia borane for hydrogen generation. RSC Advances.

[54] (2017). Nanoalumina-supported rhodium(0) nanoparticles as catalyst in hydrogen generation from the methanolysis of ammonia borane. Molecular Catalysis.

[55] (2015). Toward homogenization of heterogeneous metal nanoparticle catalysts with enhanced catalytic performance: soluble porous organic cage as a stabilizer and homogenizer. Journal of the American Chemical Society.

[56] (2019). Enhancing electrostatic interactions to activate polar molecules: Ammonia borane methanolysis on a Cu/Co(OH)$_{2}$ nanohybrid. Catalysis Science & Technology.

[57] (2018). Ultrahigh catalytic activity of L-proline-functionalized Rh nanoparticles for methanolysis of ammonia borane. Chemistry & Sustainability Energy & Materials.

