# Supplementary material for: Hydrotalcite framework stabilized ruthenium nanoparticles (Ru/HTaL): efficient heterogeneous catalyst for the methanolysis of ammonia-borane
Source: Turk J Chem. 2020 Apr 1;44(2):364–77. doi: 10.3906/kim-1910-44 (PMC7671226; doi:10.3906/kim-1910-44)
Supplement: Supplementary file 1 — Supplementary Materials [file turkjchem-44-364-sup001.pdf]

Supporting Information

Hydrotalcite framework stabilized ruthenium nanoparticles (Ru/HTaL): Efficient heterogenous catalyst for the methanolysis of ammonia-borane

İsmail Burak BAĞUÇ, Mehmet YURDERİ*, Gülşah SAYDAN KANBEROĞLU*, Ahmet BULUT

Department of Chemistry, Faculty of Science, Van Yüzüncü Yıl University, Van, Turkey

*Correspondence: mehmet.yurderi@yahoo.com, gskanberoglu@yyu.edu.tr

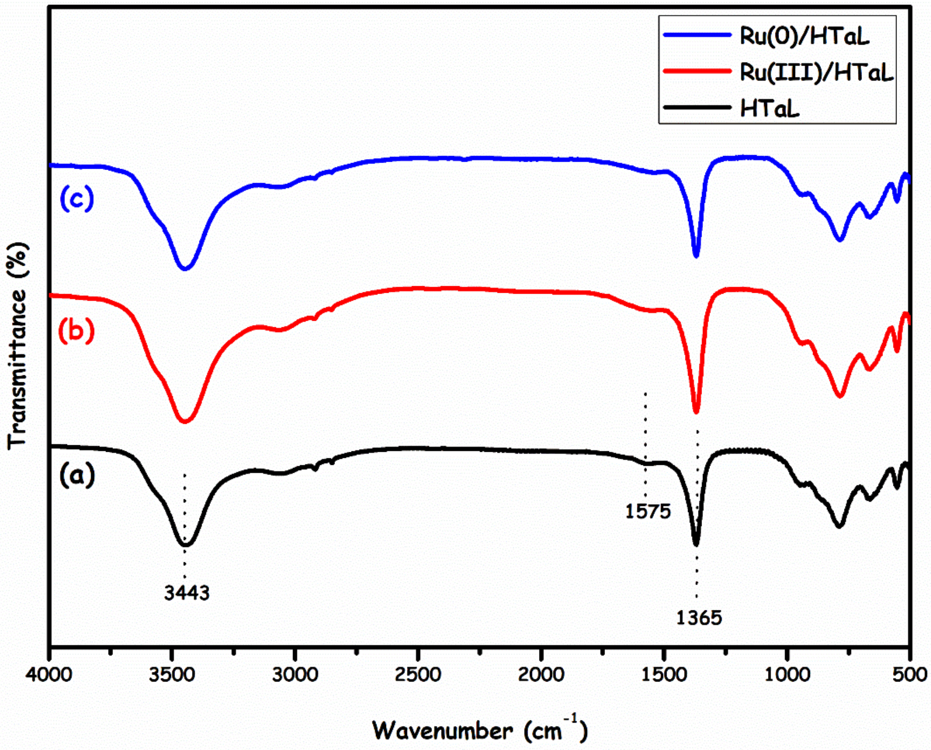

Figure S1. FTIR spectra of (a) HTaL (b) Ru(III)/HTaL and (c) Ru(0)/HTaL samples in the range of 500-4000 cm^{-1}.

Figure S1 shows the Fourier transform infrared spectroscopy (FTIR) spectra of HTaL, Ru(III)/HTaL and Ru(0)/HTaL. As can be seen in the FTIR spectrum, the absorption band (in both Ru(0)/HTaL and HTaL) at 3443 cm^{-1} is assigned to the vibrational absorption of O-H (hydroxide), which can be attributed to the interlayer water molecules and the -O-H groups in the brucite-like layers. The weak peak at 1575 cm^{-1} is assigned to the vibrational absorption coming from the interlayer water. The strong peak at 1365 cm^{-1} is assigned to the asymmetric stretching of the CO_3^{-2} (carbonate). The bands in the range of 1030-500 cm^{-1} are attributed to metal oxide (RuO_2, Al_2O_3, and MgO) stretching.

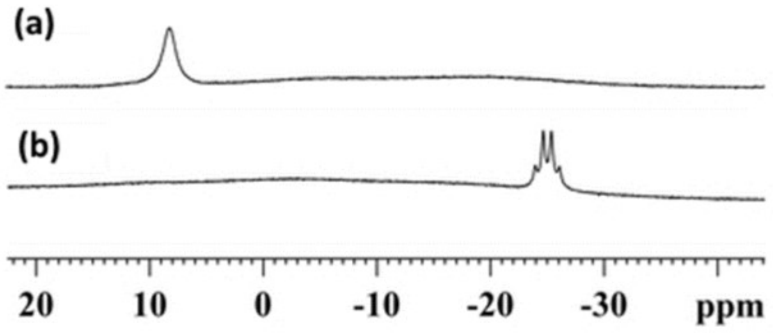

Figure S2. ^{11}B NMR spectra of **(b)** NH_3BH_3 solution, **(a)** the aliquot taken from the reaction solution at the end of the in situgenerated Ru(0)/HTaL-catalyzed methanolysis of ammonia-borane.
